# FOXC1 is associated with estrogen receptor alpha and affects sensitivity of tamoxifen treatment in breast cancer

**DOI:** 10.1002/cam4.990

**Published:** 2016-12-28

**Authors:** Jinhua Wang, Yali Xu, Li Li, Lin Wang, Ru Yao, Qiang Sun, Guanhua Du

**Affiliations:** ^1^ The State Key Laboratory of Bioactive Substance and Function of Natural MedicinesBeijing Key Laboratory of Drug Target Research and Drug ScreenInstitute of Materia MedicaChinese Academy of Medical Science and Peking Union Medical CollegeBeijing100050China; ^2^ Department of Molecular OncologyJohn Wayne Cancer Institute (JWCI) at Providence Saint John's Health CenterSanta MonicaCalifornia90404; ^3^ Department of Breast SurgeryPeking Union Medical College HospitalPeking Union Medical CollegeChinese Academy of Medical SciencesBeijing100032China

**Keywords:** Breast cancer, estrogen receptor, FOXC1, TCGA, triple negative

## Abstract

FOXC1 is a member of Forkhead box transcription factors that participates in embryonic development and tumorigenesis. Our previous study demonstrated that FOXC1 was highly expressed in triple‐negative breast cancer. However, it remains unclear what is the relation between FOXC1 and ER*α* and if FOXC1 regulates expression of ER*α*. To explore relation between FOXC1 and ER*α* and discover regulation of ER*α* expression by FOXC1 in breast cancer, we analyzed data assembled in the Oncomine and TCGA, and found that there was significantly higher FOXC1 expression in estrogen receptor‐negative breast cancer than that in estrogen receptor‐positive breast cancer. Overexpression of FOXC1 reduced expression of ER*α* and cellular responses to estradiol (E2) and tamoxifen in the MCF‐7 FOXC1 and T47D FOXC1 cells, while knockdown of FOXC1 induced expression of ER*α* and improved responses to estradiol (E2) and tamoxifen in BT549 FOXC1 shRNA and HCC1806 FOXC1 shRNA cells. In addition, overexpression of FOXC1 reduced expression of progesterone receptor (PR), Insulin receptor substrate 1 (IRS1), and XBP1 (X‐Box Binding Protein 1) and significantly reduced luciferase activity caused by E2 using ERE luciferase reporter assay. These results suggested that FOXC1 regulated expression of ER*α* and affected sensitivity of tamoxifen treatment in breast cancer, and that FOXC1 may be used as a potential therapeutic target in ER*α*‐negative breast cancer.

## Introduction

Breast cancer is the most frequently diagnosed cancer in women 1. Breast cancer is the leading cause of cancer mortality in women worldwide resulting in more than 500,000 deaths. Estrogen receptor alpha (ER*α*) plays an important role in mammal normal physiological functions and is also intensively related to pathogenesis of breast cancer 2. ER*α* expression defines a subset of cancer patients who, in general, have a better prognosis than patients with ER*α*‐negative tumor 3, 4.

Estrogen receptor is considered as an important therapeutic target as positive ER expression defines better prognosis in patients with breast cancer. ER is a ligand‐inducible transcription factor that belongs to the nuclear receptor superfamily and a key regulatory molecule in mammary epithelial cell development. Recently, a lot of researches were focused on regulation of ER*α* 5, 6. Microarray analyses and experiments have revealed that expression of forkhead box A1 (FOXA1) and GATA‐binding protein 3 (GATA‐3) are closely associated with ER*α* and they encode transcription factors which potentially involve in the ER*α*‐mediated action in breast cancer 7, 8.

FOX (Forkhead box) proteins are a family of transcription factors that play important roles in regulating the expression of genes involved in cell growth, proliferation, differentiation, and longevity. Many FOX proteins are important to embryonic development and play important roles in tumorigenesis 9. Recently, roles of FOX proteins in breast cancer attracted more and more attention. FOXC2 was correlated with human breast cancers and played a critical role in promoting invasion and metastasis 10. FOXA1 was a marker of luminal cells in mammary 11, 12. Downregulation of FOXM1 led to inhibition of proliferation, migration, and invasion of breast cancer cells through the modulation of extracellular matrix degrading factors 13, 14. Basal‐like breast cancers (BLBCs) underexpress estrogen receptor (ER), progesterone receptor (PR), and human epidermal growth factor receptor 2 (HER2) and encompass 60–90% of triple‐negative (ER−/PR−/HER2−) breast cancers. Our previous results showed that FOXC1 was the only gene overexpressed in BLBC consistently and exclusively, associated with poor overall survival. However, it remains unclear what relation between FOXC1 and ER*α* is and if FOXC1 regulates expression of ER*α* 15.

In this study, we performed silicon analysis using database from Oncomine (www.oncomine.org) and TCGA (The Cancer Genome Atlas) database, and showed that FOXC1 expression was higher in ER*α*‐negative breast cancers than ER*α*‐positive breast cancers. In addition, overexpression of FOXC1 reduced expression of PR, IRS1, and XBP1 (downstream target of ER*α*) and significantly reduced luciferase activity caused by E2 using ERE luciferase reporter assay. FOXC1 expression reduced stimulatory growth effect by E2 and inhibited sensitivity of cells to treatment of tamoxifen. All results indicated that FOXC1 regulated expression of ER*α* and affected sensitivity of tamoxifen treatment in breast cancer, and that FOXC1 may be used as a new therapeutic target in ER*α*‐negative breast cancer.

## Materials and Methods

### Cell culture

Human breast cancer cell lines (MCF‐7, T47D, BT549, and HCC1806) were purchased from the American Type Culture Collection (ATCC) and the cells were grown in Dulbecco's Modified Eagle's Medium (DMEM) supplemented with 10% fetal calf serum, 100U/mL penicillin, and 100 *μ*g/mL streptomycin at 37°C humidified incubator containing 5% CO2. Human primary breast cancer cell lines were obtained from Peking Union Medical College Hospital.

### Tumor specimens

Approval for the use of human tissues was approved by the Institutional Review Board (IRB) at the Peking Union Medical College Hospital, Beijing, China. Analysis was conducted on paraffin‐embedded archival tissue (PEAT) specimens of breast cancer diagnosed at the Peking Union Medical College Hospital.

### Stable transfection

MCF‐7 and T47D cells were plated in 60‐mm dishes at 80% confluence before 24 h of transfection. FOXC1–myc–flag plasmid was transfected into the MCF‐7 and T47D cells using Lipofectamine^™^ 3000 Transfection reagent (Invitrogen, Grand Island, NY) for 24 h. The cells were then screened under 0.8 mg/mL G418 (Invitrogen) for 3 weeks. MCF‐7 and T47D cells with overexpressing FOXC1 were subcloned as MCF‐7‐FOXC1 and T47D‐FOXC1, respectively 16.

BT549 and HCC1806 cells were plated in 60 mm dishes at 80% confluence before 24 h of transfection. FOXC1 shRNAs (Sigma‐Aldrich, St. Louis, MO) were stably transfected into BT549 and HCC1806 cells which have high FOXC1 expression and were selected in 5 *μ*g/mL puromycin. BT549 and HCC1806 cells which have low FOXC1 expression were subcloned as BT549 FOXC1 shRNA and HCC1806 FOXC1 shRNA, respectively.

Expression of FOXC1 was verified by Western blot analysis with anti‐FOXC1 antibody (Cata No. sc21394, Santa Cruz Biotechnology, Santa Cruz, CA), antimyc antibody (Cata No. 06‐340; EMD Millipore, San Diego, CA), and antiflag antibody (Cata No. TA50011‐100, Origene, Rockville, MD).

### Transient transfection assay

Cells were plated in 60 mm dishes at 80% confluence before 24 h of transfection. The FOXC1–myc–flag plasmid and promoter luciferase (ERE‐Luc) were transfected into MCF‐7 cells using Lipofectamine^™^ 3000 Transfection reagent (Invitrogen) 17. FOXC1–myc–flag plasmid (500 ng), Renilla (50 ng), and luciferase reporter construct (100 ng) were transfected into the cells in 6‐well plate. Renilla expression vector was cotransfected as an internal control. After transfected for 24–36 h, cells were washed twice with PBS buffer and harvested in 200 *μ*L of 1× reporter lysis buffer (Promega, Madison, WI). Cell lysis was centrifuged at 12,000 *g* for 10 min at 4°C and supernatant was collected. Cell extract (20* μ*L) was mixed with 100* μ*L Luciferase Assay Reagent (Promega, Madison, WI) at room temperature and immediately placed in GloMax^®^ —Multi diction system (Promega).

### Immunoblot analysis

Whole cell extracts were prepared from MCF‐7 vector and MCF‐7‐FOXC1, T47D vector and T47D‐FOXC1, BT549 vector and BT549 FOXC1 shRNA, and HCC1806 vector and HCC1806 FOXC1 shRNA cells. Western blot assays were done as previously described 18. Immunoblotting was done with polyclonal antibodies against FOXC1, IRS1 (insulin receptor substrate 1) (1:200; Cata No. sc7200, Santa Cruz Biotechnology), monoclonal antibodies against ER*α* (1:500, Cata No. 8644, Cell Signaling, Danvers, MA), PR (progesterone receptor) (1:500; Clone PgR 363, Dako, Carpinteria, CA), and XBP1 (Cata No, SAB2102720; Sigma‐Aldrich). Anti‐*β* actin (Cata No.A5316; Sigma‐Aldrich) was used at a 1:10000 dilution. Incubation with primary antibodies overnight was followed by incubation with secondary antibody (1:4000; Anti‐mouse IgG NA931V, Anti‐rabbit IgG NA934V GE Biosciences and 1:4000, Anti‐goat IgG, Cata No. sc2020, Biotechnology). Detection was carried out using the Pierce SuperSignal West Pico chemiluminescent substrate (Thermo fisher, Rockford, lL) followed by scanning using a Fluorchem 5500 chemiluminescence imager (Alpha Innotech Corp, San Leandro, CA).

### Real‐time reverse transcription PCR

Total RNA was isolated from MCF‐7 vector and MCF‐7‐FOXC1, T47D vector and T47D–FOXC1 cells, BT549 vector and BT549 FOXC1 shRNA, and HCC1806 vector and HCC1806 FOXC1 shRNA using RNeasy mini kit (Qiagen, Hilden, Germany), with on‐column DNase treatment to remove contaminating genomic DNA. Real‐time reverse transcription PCR (RT‐PCR) was done as in reference 19. The primers (Integrated DNA Technologies, Inc., Coralville, IA) for RT‐PCR were listed in Table S1.

### Immunocytofluorescence assay

MCF‐7 cells were transiently transfected with FOXC1‐GFP plasmid. After transfected for 24 h, the cells were digested with trypsin and mixed with untransfected MCF‐7 cells. The mixed cells were cultured in chamber slides (Nunc Lab‐Tek, St. Louis, MO). Cells were fixed with 4% formaldehyde and then permeabilized with PBS containing 0.1% Triton X‐100. Slides were blocked by 5% BSA for 30 min and incubated with a primary antibody (Cata No. 8644, anti–ER*α* antibody, 1:100, Cell Signaling, Danvers, MA) at room temperature for 1 h. Then, cells were incubated with an Alexa 546–conjugated secondary antibody (Cata No. A‐11030, 1:500, Invitrogen, Grand Island, NY) for 30 min. Slides were washed by PBS three times, 5 min each time, mounted with DAPI (Vector Laboratories, Burlingame, CA), and observed under Nikon microscope (Nikon, Melville, NY) 20.

### Cell proliferation assay

The Promega CellTiter 96^®^ AQu_eous_ One Solution Cell Proliferation Assay was used according to the manufacturer's instructions (Promega, Madison, WI). Cells were seeded into 96‐well plates (1000 cells per well) in triplicate. Absorbance at 490 nm was measured after the addition of 20 *μ*L of MTS reagent per well for 2 h, every 24 h over a 96 h period 21, 22.

### Immunohistochemistry

Five‐micrometer paraffin‐embedded tissue sections were deparaffinized and rehydrated, antigens were retrieved, and IHC was performed using an optimized protocol 23, 24. Slides were deparaffinized, rehydrated, and washed in 1X PBS. Antigen retrieval was performed with 1X citrate buffer (Sigma‐Aldrich) at 100°C for 10 min and then incubated in H_2_O_2_ (Sigma‐Aldrich) at room temperature to block endogenous peroxidase. Separate slides were incubated in primary rabbit Anti‐FOXC1 antibody (aa250‐300) IHC‐plus^™^ LS‐B1800 (1:250 dilution; Seattle, WA) overnight in a 4°C humid chamber followed by 1 h incubation with secondary biotinylated link Ab. The reaction for FOXC1 was developed using a labeled streptavidin biotin (LSAB) method (LSAB+ Kit; Dako, Carpinteria, CA) and visualized using VIP Substrate Kit (Vector Laboratories, Burlingame, CA). Specificity of the immunostaining was determined by the inclusion of isotype‐specific IgG (Santa Cruz Biotechnology, Santa Cruz, CA) as negative controls. The sections were counterstained with hematoxylin (Sigma‐Aldrich). A photograph of each IHC‐stained section was taken for analysis using a Nikon Eclipse Ti microscope and NIS elements software (Nikon, Melville, NY). Staining density was determined by Image J software (http://rsbweb.nih.gov/ij/). After adjustment for background on each selected field, the density of the individual breast cancer specimen was quantified and given a numerical value from 0 to 255. Breast cancer specimens were tested in duplicate, and the average of the two staining intensity values was used for statistical analysis.

### Nude mice for xenograft assays

Twelve nude mice (BALB/c background) (experimental animal center, Chinese Academy of Sciences, Shanghai, China) were randomly divided into two groups and housed in air conditioned, light‐controlled, animal facilities. Animal care and all experiments were in accordance with the institutional guidelines and were approved by the Animal Care and Use Committee in accordance with regulations of Institutional Animal Care and Use Committee. To test the tumorigenic properties of cells, MCF‐7 vector and MCF‐7 FOXC1 (1 × 10^6^) cells were orthotopically injected into the number four mammary fat pads of female nude‐BALB/c mice (6 mice for each cell line) 25.

Mice were weighed and subcutaneous tumors were measured after a week; tumor volume was obtained by the ellipsoid volume calculation formula: 0.5× (length × width^2^).

### Statistical analysis

The results are given as mean ± SD of samples measured in triplicate. Each experiment was repeated three times, unless otherwise indicated. Student's *t*‐test was used to calculate differences between the various study groups. The difference was considered statistically significant at *P *<* *0.05.

## Results

### FOXC1 is negatively correlated with luminal expression signatures: ESR1, FOXA1, GATA3, XBP1, and MYB

**Figure 1:**
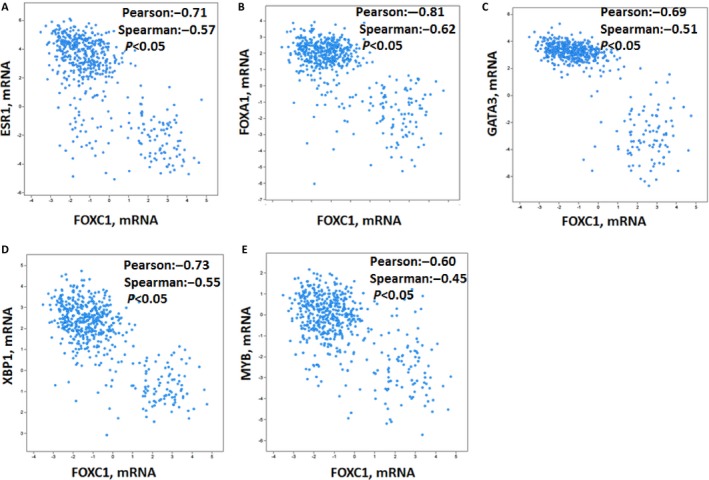
FOXC1 is negatively correlated with luminal expression signatures. (A) FOXC1 is negatively correlated with ESR1. (B) FOXC1 is negatively correlated with FOXA1. (C) FOXC1 is negatively correlated with GATA3. (D) FOXC1 is negatively correlated with XBP1. (E) FOXC1 is negatively correlated with MYB.

Our previous study showed that FOXC1 was highly expressed in triple‐negative breast cancer. However, the mechanism remains unclear. To explore the mechanism, silicon assay was performed using TCGA database (http://www.cbioportal.org/public-portal/cross_cancer.do). Results showed that expression of FOXC1 mRNA was negatively correlated with luminal expression signatures: ESR1, FOXA1, GATA3, XBP1, and MYB expression (Fig.**** 1A–E). We also found that FOXC1 was amplified in invasive breast cancer (8%) (Fig. S1**).**

### FOXC1is highly expressed in ER*α*‐negative breast cancer

**Figure 2:**
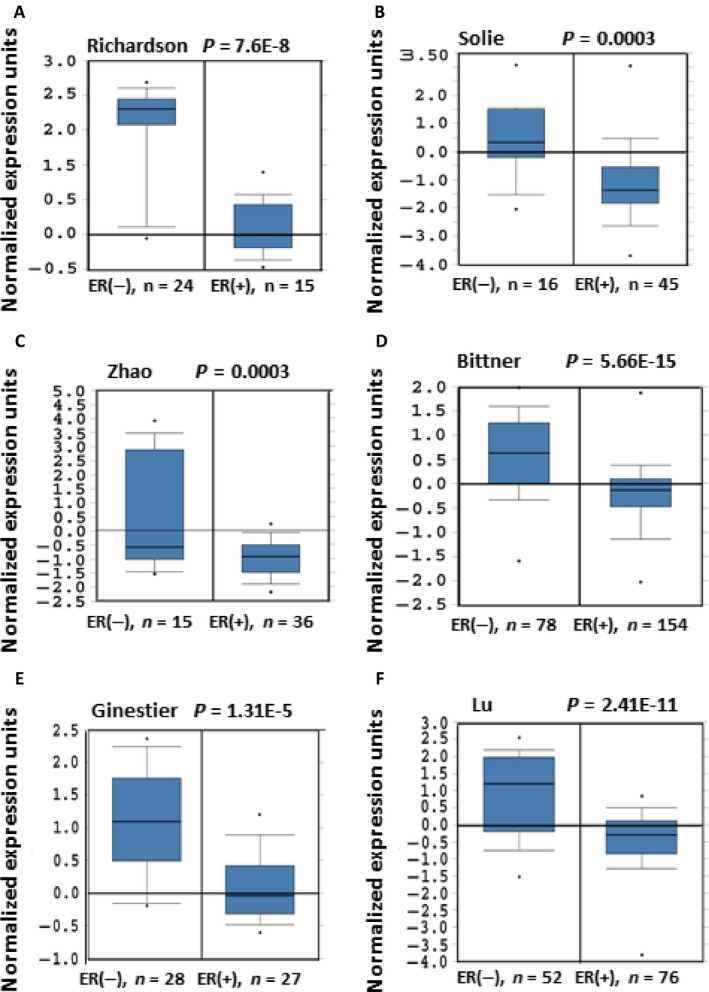
FOXC1 expression is negatively related with ER*α* expression. (A–F) There was higher FOXC1 expression in ER*α*‐negative tumors than that in ER*α*‐positive tumors. Data and statistics were obtained from www.oncomine.org (Richardon et al., 2006; Sorlie et al., 2001; Zhao et al., 2004; Bittner et al., 2001; Gluck et al., Ginestier et al., 2006; 2011; Lu et al., 2008).

Basal‐like breast cancers are characterized by high expression of basal cytokeratins, low or absent expression of estrogen receptor, progesterone receptor, and HER2. Our previous study showed that there was high FOXC1 expression in basal‐like breast cancers (no or very low ER*αα* expression). To confirm the relation between FOXC1 expression levels and ER*α* status of breast cancer, we compared the expression levels of FOXC1 in the ER*α*‐negative and ‐positive breast cancer using the Oncomine and TCGA (The Cancer Genome Atlas) database, which provides publicly available datasets on cancer gene expression. Eleven of the 11 datasets from Oncomine, which contain gene chip profiles classified as normal or breast carcinoma tissues, showed that FOXC1 mRNA levels were much higher in ER*α*‐negative breast cancers than that in ER*α*‐positive breast cancers (Fig.**** 2 and S2). TCGA data also confirmed the result (Fig. S3**)**.

### FOXC1 reduced expressions of ER*α* and its downstream targets

**Figure 3:**
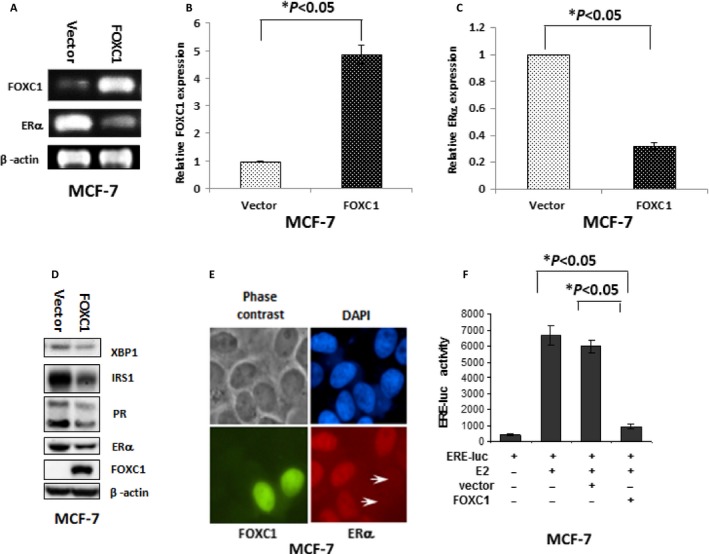
FOXC1 reduced expression of ER*α* and its downstream targets. (A) ER*α* mRNA expression was decreased when FOXC1 mRNA expression was increased. (B) Quantification of FOXC1 mRNA expression in MCF‐7 vector and MCF‐7‐FOXC1. (C) Quantification of ER*α* mRNA expression in MCF‐7 vector and MCF‐7‐FOXC1. (D) Protein expression of PR, IRS‐1, and XBP1 (downstream target of ER*α*) was decreased when FOXC1 protein expression was increased. (E) There is almost no ER*α* expression in cells which have high FOXC1 expression level (green color) transfected by FOXC1 expression plasmid. (F) FOXC1 efficiently reduced ER*α* transcription in MCF‐7 breast cancer cells. PR, progesterone receptor.

FOXC1 is the only gene to be consistently and exclusively overexpressed in BLBC and is associated with poor overall survival (*P* = 0.0001) (11). To test whether FOXC1 affects ER*α* gene expression, MCF‐7 and T47D were transfected with FOXC1–myc–flag plasmid, and the stable clones were obtained. All these clones were pooled. We carried out real‐time PCR and Western blot assay by using the MCF‐7 vector and MCF‐7‐FOXC1, T47D vector and T47D‐FOXC1. There is much lower ER*α* mRNA and protein expression in the MCF‐7‐FOXC1 and T47D‐FOXC1 cells than that in the MCF‐7 vector and T47D vector, respectively. (Fig.**** 3A–C and S4).

To investigate functional effects of the downregulation of ER*α* by FOXC1, expression of XBP1, PR, and IRS1 was evaluated as downstream targets of ER*α* using Western blot. Expression of XBP1, PR, and IRS1 was decreased in the MCF‐7‐FOXC1 and T47D‐FOXC1 cells compared to MCF‐7 vector and T47D vector, respectively (Fig.**** 3D and S5**A**).

In immunocytofluorescence assay, it was showed in Figure 3E that there is much lower ER*α* expression in transiently transfected MCF‐7 cells with FOXC1–EGFP (Enhanced Green Fluorescent Protein) plasmid than MCF‐7 vector cells. Luciferase reporter plasmids‐containing EREs were transfected into MCF‐7 cells with or without FOXC1–myc–flag. Estrogen treatment enhanced the transcription of these reporter plasmids. The estrogen‐stimulated enhanced transcription was inhibited by FOXC1 expression (Fig. 3F).

**Figure 4:**
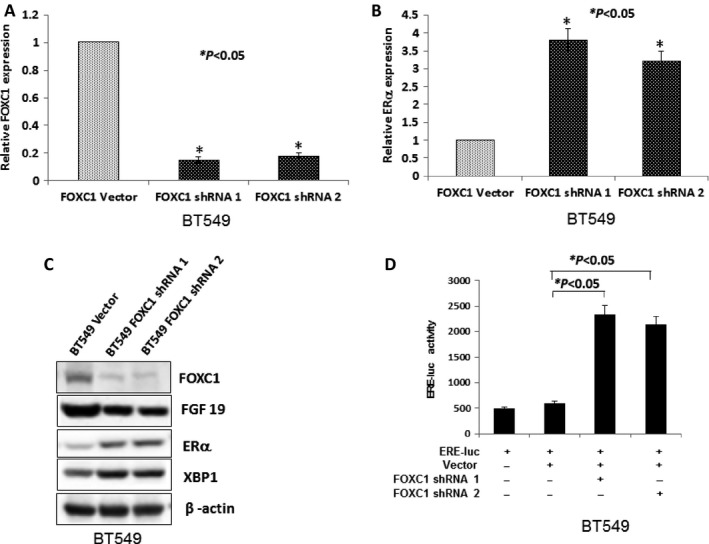
Kncokdown of FOXC1 induced expression of ER*α*. (A) Expression of FOXC1 mRNA was significantly reduced by two‐pair FOXC1 shRNAs in BT549 FOXC1 shRNA. (B) Expression of ER*α* mRNA was induced when expression of FOXC1 was reduced in BT549 FOXC1 shRNA. (C) Protein expression of ER*α* and XBP1 (downstream target of ER*α*) was increased while protein expression of FOXC1 and FGF19 (downstream target of FOXC1) was reduced in BT549 FOXC1 shRNA. (D) FOXC1 shRNA can activate ERE‐luc luciferase.

Meanwhile, BT549 and HCC1806 cell lines which have high FOXC1expression were transfected by FOXC1 shRNA and the stable clones were obtained. Expression of FGF19 (downstream target FOXC1), ER*α* and XBP1 (downstream target of ER*α*) was checked in these clones using Western blot. Results showed that mRNA expression of ER*α* was increased when mRNA expression of FOXC1 was reduced by FOXC1 shRNA **(**Figs. 4A, B and S6A, B**)**. Expression of ER*α* and XBP1 was increased when expression of FOXC1 and FGF19 was reduced in BT549 FOXC1 shRNA and HCC1806 FOXC1 shRNA **(**Fig. 4C, S6C). FOXC1 shRNA activated ERE‐luc activity in BT549 and HCC1806 cell lines (Figs. 4D and S6D).

Taken together, these results showed that FOXC1 regulated the expression of ER*α*

### FOXC1 reduced cell response to estrogen and tamoxifen

ER*α* belongs to the steroid hormone receptor superfamily and functions as a ligand‐activated transcription factor. ER*α* is a key player in estrogen‐mediated proliferation, survival, and differentiation through regulating the transcription of estrogen‐target genes as well as through activation of signal transduction pathways 26.

**Figure 5:**
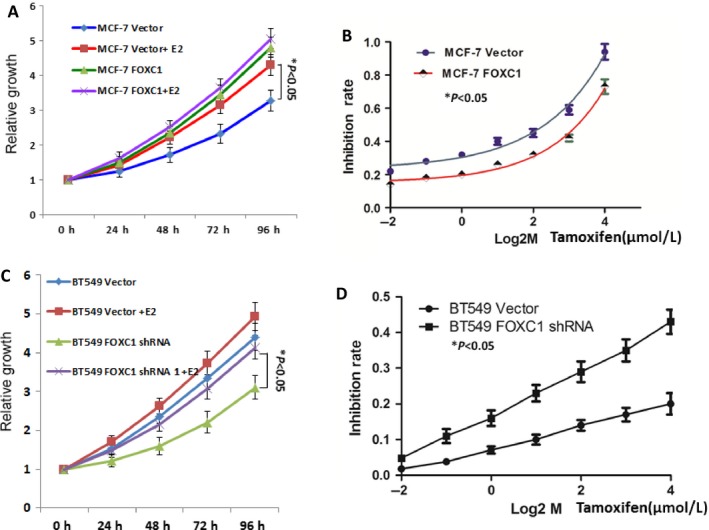
FOXC1 reduced cell response to estrogen and tamoxifen. (A) Growth of MCF‐7‐FOXC1 was not more sensitive to E2 than that of MCF‐7 vector. (B) MCF‐7‐FOXC1 was not more sensitive to treatment of tamoxifen than MCF‐7 vector. (C) Growth of BT549 FOXC1 shRNA was more sensitive to E2 than that of BT549 vector. (D) BT549 FOXC1 shRNA was more sensitive to treatment of tamoxifen than BT549 vector.

To investigate the relation between FOXC1 and ER*α* in the growth of breast cancer cells, MCF‐7 vector and MCF‐7‐FOXC1, T47D vector and T47D‐FOXC1, BT549 vector and BT549 FOXC1 shRNA, HCC1806 vector and HCC1806 FOXC1 shRNA cells were cultured in DMEM (without phenol red) with E2 or normal culture medium. FOXC1 expression reduced stimulatory growth effect by E2 in MCF‐7‐FOXC1 and T47D‐FOXC1 compared to MCF‐7 vector and T47D vector in DMEM (without phenol red) with E2, respectively, while the growth of BT549 FOXC1 shRNA and HCC1806 FOXC1 shRNA cells was higher than BT549 vector and HCC1806 vector in DMEM (without phenol red) with E2, respectively. (Figs.**** 5A, C and S7A, C).

To explore whether the FOXC1 renders resistance to the tamoxifen in cells, MCF‐7 vector, MCF‐7‐FOXC1, T47D vector, and T47D‐FOXC1 cells were cultured in DMEM (without phenol red) with tamoxifen. As shown in Figures 5B and S7B, MCF‐7‐FOXC1 and T47D‐FOXC1 cells became more resistant to tamoxifen than control cells.

We also check sensitivity of tamoxifen treatment in BT549 FOXC1 shRNA and HCC1806 FOXC1 shRNA and found that BT549 FOXC1 shRNA and HCC1806 FOXC1 shRNA cells were more sensitive to tamoxifen than BT549 vector and HCC1806 vector cells, respectively **(**Figs. 5D and S7D).

### FOXC1 was overexpressed in ER*α*‐negative breast cancer cell lines

**Figure 6:**
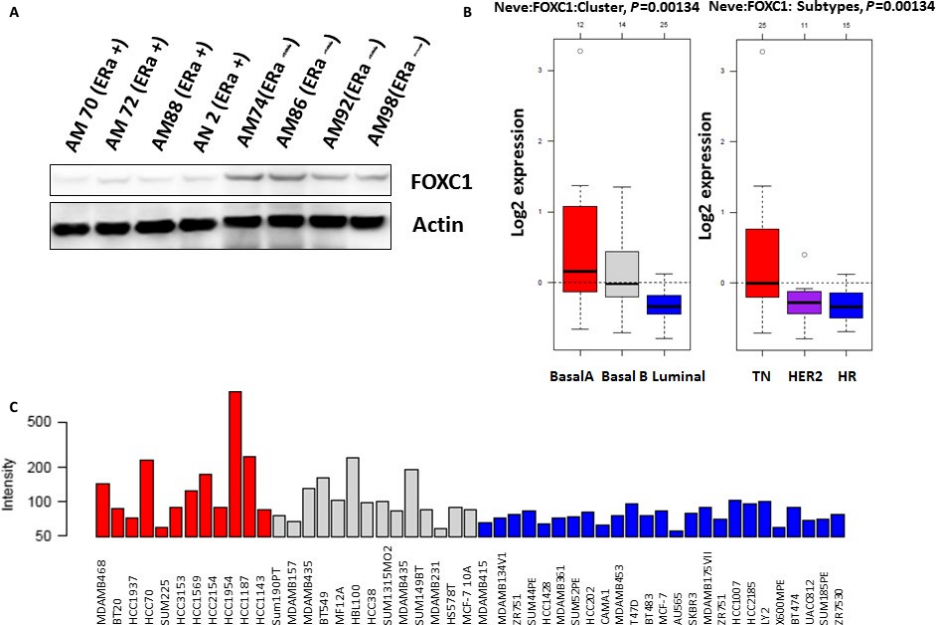
FOXC1 was highly overexpressed in ER*α*‐negative breast cancer cell lines. (A) FOXC1 expression was much higher in ER*α*‐negative primary breast cell lines (AM70, AM72, AM88, and AN2) than that in ER*α*‐positive primary breast cell lines (AM74, AM86, AM9,2 and AM98). (B–C) FOXC1 expression in triple‐negative breast cancer cells was significantly higher than that in HER 2‐positive or luminal (ER*α*‐positive) breast cancer cell lines. ER*α* (+): ER*α* positive; ER*α* (‐): ER*α* negative.

To check if FOXC1 expression is related with status of ER*α* in breast cancer cell lines, Western blot was performed using cell lysis from cell lines of primary breast cancers. FOXC1 expression was much higher in ER‐negative cell lines than that in ER‐positive cell lines (Fig. 6A). Silicon assay (http://co.bmc.lu.se/gobo/) showed that FOXC1 expression in triple‐negative cells was significantly higher than that in HER 2‐positive and luminal cell lines (Figs. 6B, C and S8). All results suggested that FOXC1 was overexpressed in ER*α*‐negative breast cancer cell lines.

### FOXC1 was overexpressed in tissues of ER*α*‐negative breast cancer

**Figure 7:**
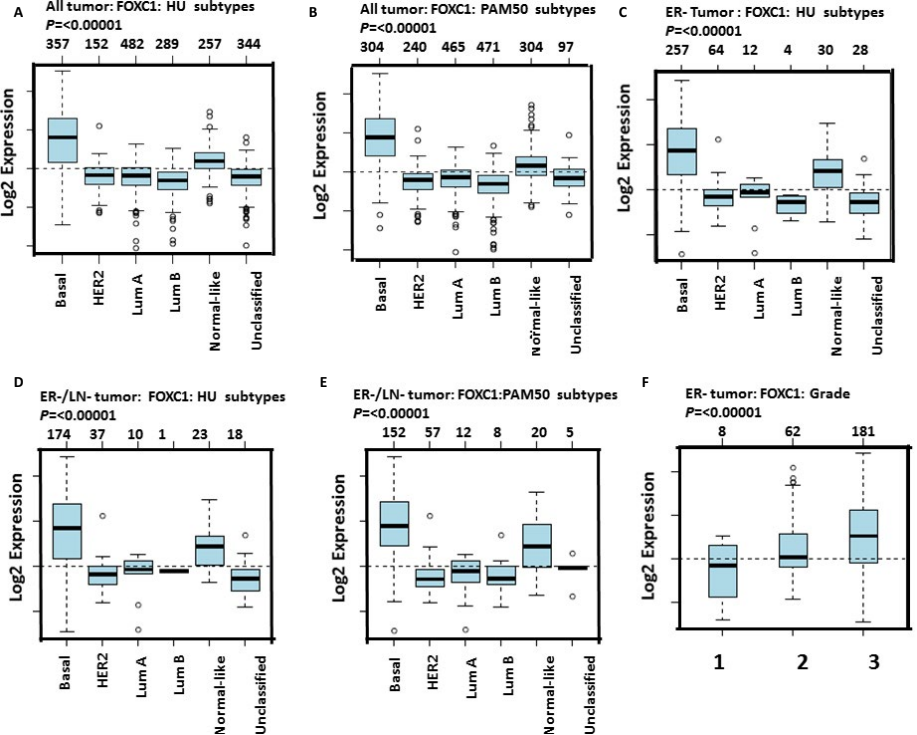
FOXC1 mRNA was overexpressed in tissues of ER*α*‐negative breast cancer breast cancer. (A–E) Expression of FOXC1 in basal‐like breast cancer (ER*α*‐negative breast cancer) was higher than other types of breast cancer. (F) Expression of FOXC1 was increased as progress of ERα‐negative breast cancer.

**Figure 8:**
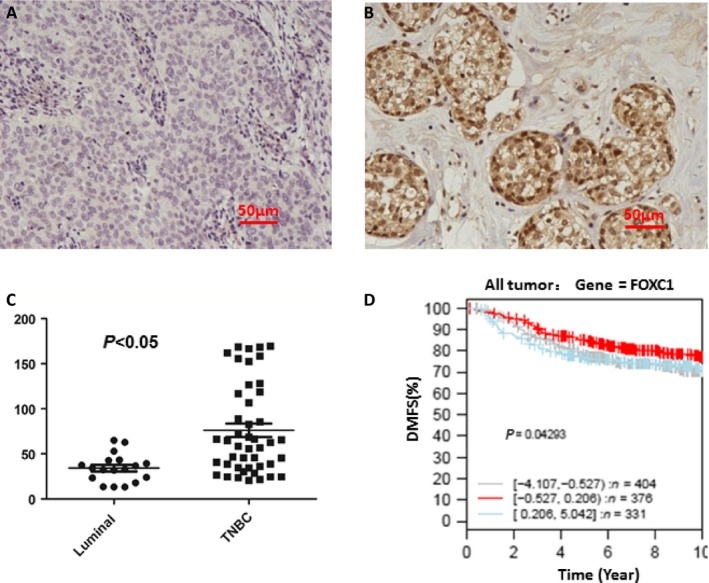
FOXC1 protein was overexpressed in tissues of ER*α*‐negative breast cancer. (A) The representative photographs were shown; negative control (Left), positive (Right). (B) IHC results showed a significantly higher FOXC1 expression in triple‐negative breast cancer (*N* = 53) compared to luminal breast cancer (*N* = 19). (C) The patients with high FOXC1 expression have the lowest survival rate.

**Figure 9:**
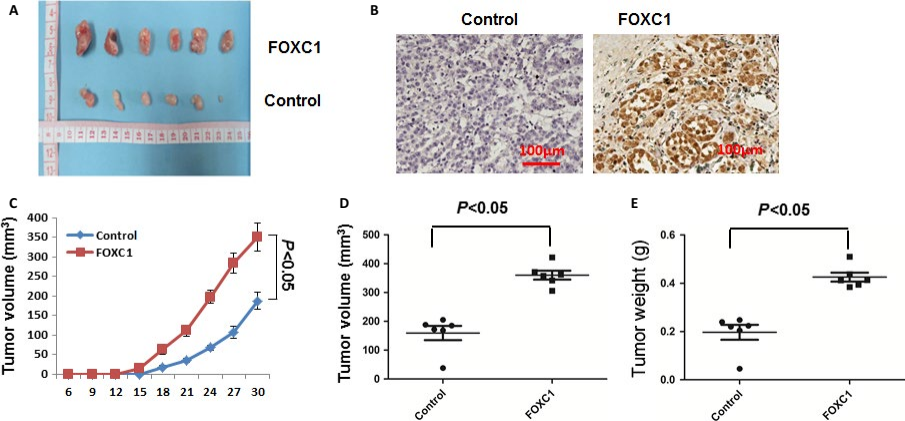
Growth of tumor xenografts in nude mice: comparison of MCF‐7 vector and MCF‐7‐FOXC1. (A) Tumor xenografts in MCF‐7‐FOXC1 group are larger than that in MCF‐7 vector group. (B) Representative IHC photographs of FOXC1 expression in xenograft tumor. (C) Growth curve of xenograft tumors in MCF‐7‐FOXC1 group and MCF‐7 vector group. Growth of xenograft tumors from MCF‐7‐FOXC1 cells is higher than that of xenograft tumors form MCF‐7 vector cells. (D) Volume of xenograft tumor in MCF‐7‐FOXC1 group and MCF‐7 vector group. Volume of xenograft tumor in MCF‐7‐FOXC1 group is more than that in MCF‐7‐FOXC1 vector group. (E) Final weight of xenograft tumors in MCF‐7‐FOXC1 group and MCF‐7 vector group. Final weight of xenograft tumors in MCF‐7‐FOXC1 group is more than that in MCF‐7‐FOXC1 group.

To check FOXC1 expression in breast cancer, we firstly analyzed the GOBO online database (http://co.bmc.lu.se/gobo/), which consists of 10 breast cancer cohorts and 1789 patients. It was shown in Figure**** 7A–E that expression of FOXC1 in basal‐like breast cancer was higher than other type breast cancer. Data also showed that there was higher FOXC1 expression in ER*α*‐negative tumor than that in ER*α*‐positive tumor (Fig.  9)**.** Expression of FOXC1 was increased with progress of breast cancer (Fig. 7F and S10). Secondly, Immunohistochemistry (IHC) was done to assess the expression of protein in tissues of human breast cancer. The representative pictures were shown in Figure 8 A and B. Results from IHC showed there was a significantly higher FOXC1 expression in triple‐negative breast cancer compared to luminal breast cancer (Fig.**** 8 **C).**

To assess the clinical significance of FOXC1 in breast cancer, Kaplan–Meier meta‐analyses were performed using the GOBO online database (http://co.bmc.lu.se/gobo). Patients with high FOXC1 expression have the lowest survival rate **(**Fig. 8D**)**. These findings support the notion that FOXC1 plays a major role in the progression of breast cancer metastasis.

### FOXC1 enhanced growth of xenograft tumors in nude mice

To assess whether FOXC1 increased the tumorigenic properties of breast cancer cell lines, MCF‐7 vector and MCF‐7‐FOXC1 cells were orthotopically injected into the number four mammary fat pads of female nude‐BALB/c mice (6 mice/cell line). Growth and size of tumor were significantly increased in MCF‐7‐FOXC1 compared to MCF‐7 vector **(**Fig. 9A**)**. Expression of FOXC1 increased the volume and weight of tumors (Fig. 9B–E). In summary, FOXC1 increased the tumorigenic properties of breast cancer cell lines.

## Discussion

FOX (Forkhead box) proteins are a kind of important transcription factors which regulate the expression of genes related to cell growth, proliferation, differentiation, and longevity 27. Many FOX proteins are important to embryonic development. FOXC1, a member of Forkhead box transcription factors essential for mesoderm tissue development in vertebrates 28, 29, consistently showed the highest correlation with the basal‐like subtype compared to other subtypes in the breast cancer. In this study, we found that there was significantly higher FOXC1 expression in ER*α*‐negative breast cancer and ER*α*‐positive breast cancer. Overexpression FOXC1 decreased expression of ER*α* protein and reduced cellular responses to estradiol and tamoxifen, while knockdown of FOXC1 induced expression of ER*α* protein and improved cellular responses to estradiol and tamoxifen.

ER*α* positivity was associated with significantly better survival than patients with ER*α*‐negative tumors, which are widely believed to have a poor prognosis 30. Our previous results showed that FOXC1 was significantly upregulated in basal‐like specimens as compared to specimens of other molecular subtypes or in normal breast tissues 15. Our current data showed that there was a significant correlation between FOXC1 expression levels and ER*α* negative breast cancer and further supported previous conclusion. Overexpression of FOXC1 decreased ER*α* mRNA and protein expression. PR (progesterone receptor), IRS1 (the insulin receptor substrate 1), and XBP1 were considered as downstream targets of ER*α*. Expression of PR, IRS1, and XBP1 was also reduced when FOXC1 expression was increased. In addition, ERE‐luc activity was dramatically increased by E2 and the increase was reduced by FOXC1 expression. These results suggest that FOXC1 regulated expression of ER*α*. The recent study also showed that FOXC1 was involved in ER*α* silence 31. Our primary data showed that FOXC1 could bind promoter of ESR1 (not shown here). The exact mechanism for how FOXC1 regulated ER*α* was still not clear and more studies were needed to focus on the mechanism in the future. Breast cancer cells which expressed ER*α* are sensitive to E2 stimulation and tamoxifen treatment, such as MCF‐7 and T47D cells 6, 32, 33. The expression of ER*α* was reduced when expression of FOXC1 was increased in MCF‐7‐FOXC1 and T47D‐FOXC1, while expression of ER*α* was increased when expression of FOXC1 was reduced in BT549 FOXC1 shRNA and HCC1806 FOXC1 shRNA. This can explain why that growth of MCF‐7‐FOXC1 and T47D‐FOXC1 was not more sensitive to E2 than MCF‐7 vector and T47D vector while growth of BT549 FOXC1 shRNA and HCC1806 FOXC1 shRNA was more sensitive to E2 than BT549 vector and HCC1806 vector in DMEM (without phenol red) with E2, respectively. Similarly, MCF‐7‐FOXC1 and T47D‐FOXC1 cells are more insensitive to treatment of tamoxifen than MCF‐7 vector and T47D vector, while BT549 FOXC1 shRNA and HCC1806 FOXC1 shRNA are more sensitive to treatment of tamoxifen. Therefore, FOXC1 may be used as biomarker for treatment in breast cancers by tamoxifen.

Elevated FOXC1 mRNA expression was associated with triple‐negative breast cancers compared to other hormone receptor breast cancer. There is higher FOXC1 expression in ER*α*‐negative primary breast cell lines than that in ERα‐positive primary breast cell lines. Similar situations were seen in breast cancer tissues and cell lines. FOXC1 expression was increased as development of breast cancer. These results raise the possibility that FOXC1 and ER*α* coordinately regulate progression of breast cancer.

In summary, our study has firstly reported significant correlation between FOXC1 and ER*α* in breast cancer, which might be responsible for the poor progress of triple‐negative breast cancer in clinic. In addition, it was shown that overexpression of FOXC1 increased the tumorigenic properties of breast cancer cells. Therefore, FOXC1 is possibly to be a potential drug target for triple‐negative breast cancer.

## Conflict of Interest

Authors declare no competing financial interests in relation to the work described.

## Supporting information


**Figure S1.** FOXC1 was amplified in invasive breast cancer. TCGA data showed that there was 8% FOXC1 amplification patients in all invasive breast cancer patients (8%, 64 in 825).
**Figure S2.** FOXC1 was negatively correlated with expression of ER*α* in breast cancer. (A–E): There was higher FOXC1 expression in ER*α*‐negative tumors than that in ER*α*‐positive tumors. Data and statistics were obtained from www.oncomine.org (Pollock et al., 2006; Peou et al., 2000; Sorlie et al., 2003; Schuetz et al., 2006; Turashvili et al., 2007).
**Figure S3.** FOXC1 was highly expressed in ER*α*‐negative breast cancer. TCGA data showed there was higher FOXC1 expression in ER*α*‐negative tumors than that in ER*α*‐positive tumors.
**Figure S4.** Expression of ER*α* mRNA was reduced in T47D‐FOXC1 cells. (A) FOXC1 mRNA expression in the T47D‐FOXC1 and T47D vector cells was shown. (B) ER*α* mRNA expression in the T47D‐FOXC1 and T47D vector cells was shown. Expression of ER*α* was reduced as FOXC1 expression was increased.
**Figure S5.** FOXC1 reduced expression of ER*α*. **(**A) Expression of ER*α* and XBP1 was decreased when expression of FOXC1 was increased in T47D cells. (B) Luciferase reporter plasmids containing EREs were transfected into T47D cells with or without FOXC1–myc–flag. Estrogen treatment can activate ERE‐luc luciferase and the activation can be dramatically reduced by FOXC1 in T47D.
**Figure S6.** Knockdown of FOXC1 induced expression of ER*α*. (A) Expression of FOXC1 mRNA was significantly reduced by two‐pair FOXC1 shRNAs in HCC1806 FOXC1 shRNA. (B) Expression of ER*α* mRNA was induced when expression of FOXC1 was reduced in HCC1806 FOXC1 shRNA. (C) Protein expression of ER*α* and XBP1 (downstream target of ER*α*) was increased while protein expression of FOXC1 and FGF19 (downstream target of FOXC1) was reduced in HCC1806 FOXC1 shRNA. D. FOXC1 shRNA can activate ERE‐luc luciferase.
**Figure S7.** FOXC1 reduced cell response to estrogen and tamoxifen. (A) Growth of T47D‐FOXC1 was not more sensitive to E2 than that of T47D vector. (B) T47D‐FOXC1 was not more sensitive to treatment of Tamoxifen than T47D vector. (C) Growth of HCC1806 FOXC1 shRNA was more sensitive to E2 than that of HCC1806 vector. (D) HCC1806 FOXC1 shRNA was more sensitive to treatment of Tamoxifen than HCC1806 vector.
**Figure S8.** FOXC1 was highly expressed in triple‐negative breast cancer. FOXC1 expression in triple‐negative breast cancer cells was significantly higher than that in HER 2‐positive and luminal breast cancer cell lines.
**Figure S9.** FOXC1 was highly expressed in ER*α*‐negative tumor. There is higher FOXC1 expression in ER*α*‐negative tumors than that in ER*α*‐positive tumors.
**Figure S10.** FOXC1 expression was increased as development of breast cancer. FOXC1 expression was increased as the progress of all breast cancer.
