# Supplementary material for: FOXC1 is associated with estrogen receptor alpha and affects sensitivity of tamoxifen treatment in breast cancer
Source: Cancer Med. 2016 Dec 28;6(1):275–87. doi: 10.1002/cam4.990 (PMC5269562; doi:10.1002/cam4.990)
Supplement: Supplementary file 1 — Figure S1. FOXC1 was amplified in invasive breast cancer. TCGA data showed that there was 8% FOXC1 amplification patients in all invasive breast cancer patients (8%, 64 in 825). Figure S2. FOXC1 was negatively correlated with expression of ERα in breast cancer. (A–E): There was higher FOXC1 expression in ERα‐negative tumors than that in ERα‐positive tumors. Data and statistics were obtained from www.oncomine.org (Pollock et al., 2006; Peou et al., 2000; Sorlie et al., 2003; Schuetz et al., 2006; Turashvili et al., 2007). Figure S3. FOXC1 was highly expressed in ERα‐negative breast cancer. TCGA data showed there was higher FOXC1 expression in ERα‐negative tumors than that in ERα‐positive tumors. Figure S4. Expression of ERα mRNA was reduced in T47D‐FOXC1 cells. (A) FOXC1 mRNA expression in the T47D‐FOXC1 and T47D vector cells was shown. (B) ERα mRNA expression in the T47D‐FOXC1 and T47D vector cells was shown. Expression of ERα was reduced as FOXC1 expression was increased. Figure S5. FOXC1 reduced expression of ERα. (A) Expression of ERα and XBP1 was decreased when expression of FOXC1 was increased in T47D cells. (B) Luciferase reporter plasmids containing EREs were transfected into T47D cells with or without FOXC1–myc–flag. Estrogen treatment can activate ERE‐luc luciferase and the activation can be dramatically reduced by FOXC1 in T47D. Figure S6. Knockdown of FOXC1 induced expression of ERα. (A) Expression of FOXC1 mRNA was significantly reduced by two‐pair FOXC1 shRNAs in HCC1806 FOXC1 shRNA. (B) Expression of ERα mRNA was induced when expression of FOXC1 was reduced in HCC1806 FOXC1 shRNA. (C) Protein expression of ERα and XBP1 (downstream target of ERα) was increased while protein expression of FOXC1 and FGF19 (downstream target of FOXC1) was reduced in HCC1806 FOXC1 shRNA. D. FOXC1 shRNA can activate ERE‐luc luciferase. Figure S7. FOXC1 reduced cell response to estrogen and tamoxifen. (A) Growth of T47D‐FOXC1 was not more sensitive to E2 than that of T47D vec [file CAM4-6-275-s001.pdf]

Supplementary Figure 1

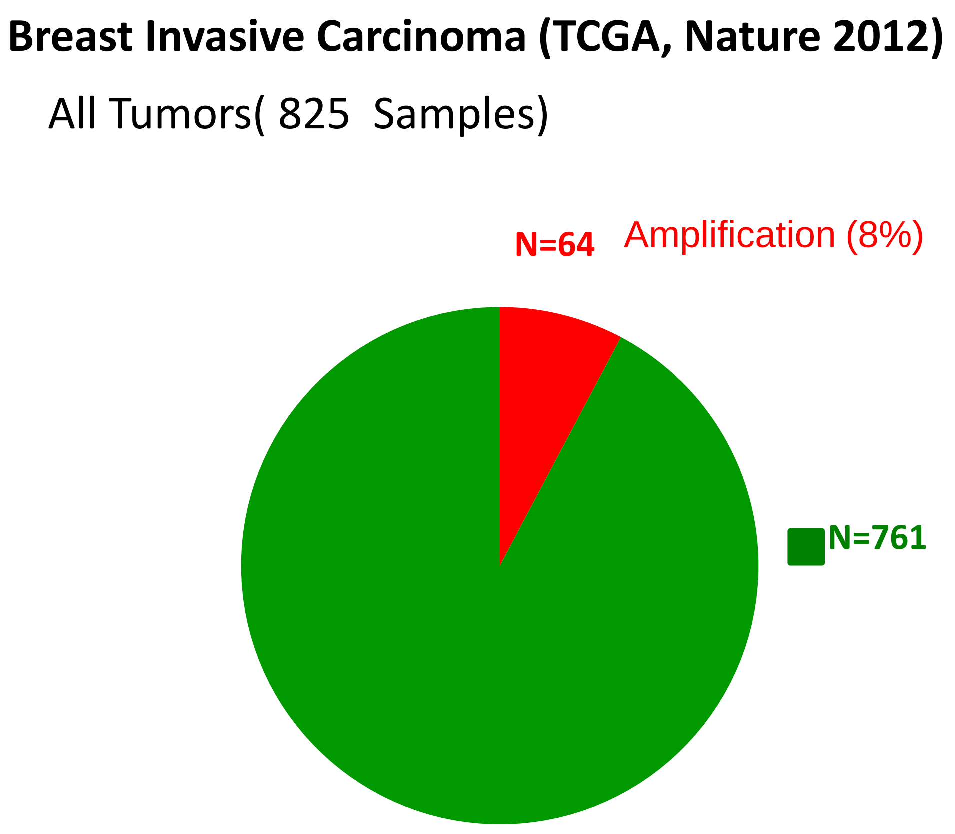

Supplementary Figure 2

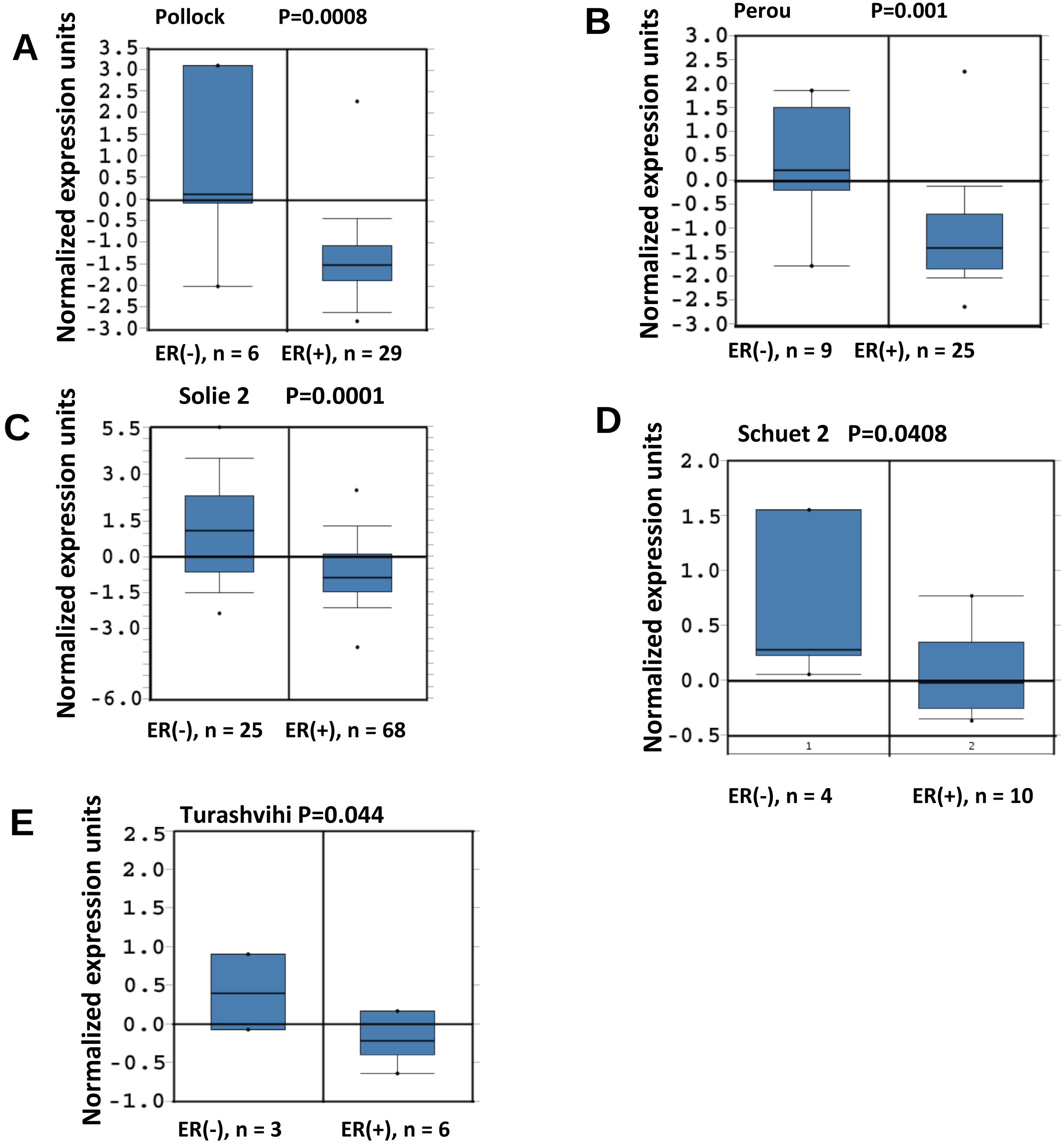

Supplementary Figure 3

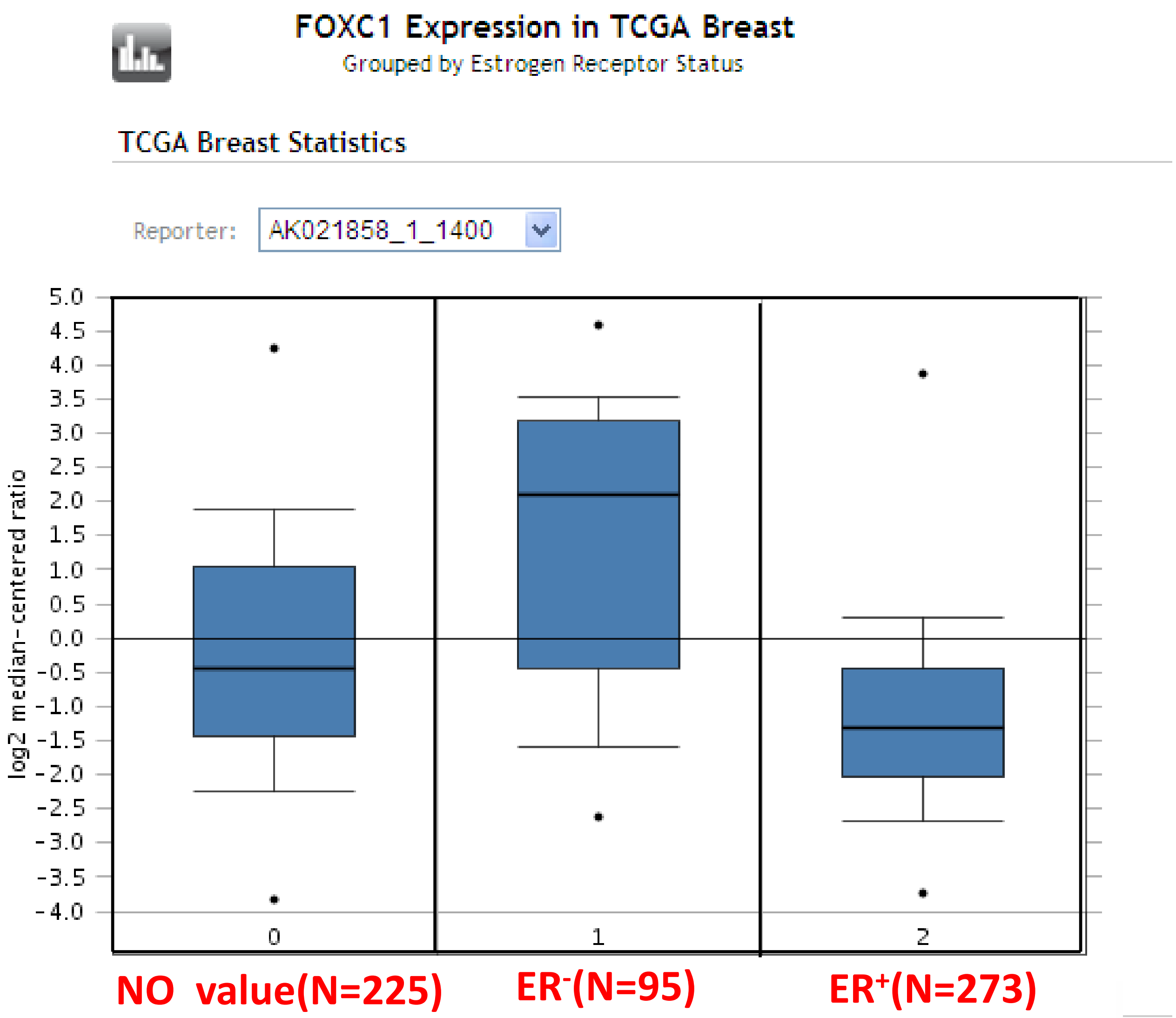

Supplementary Figure 4

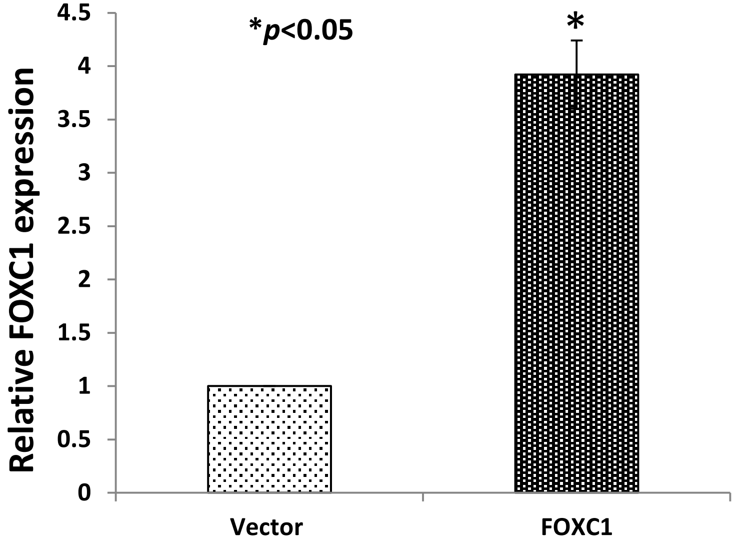

T47D

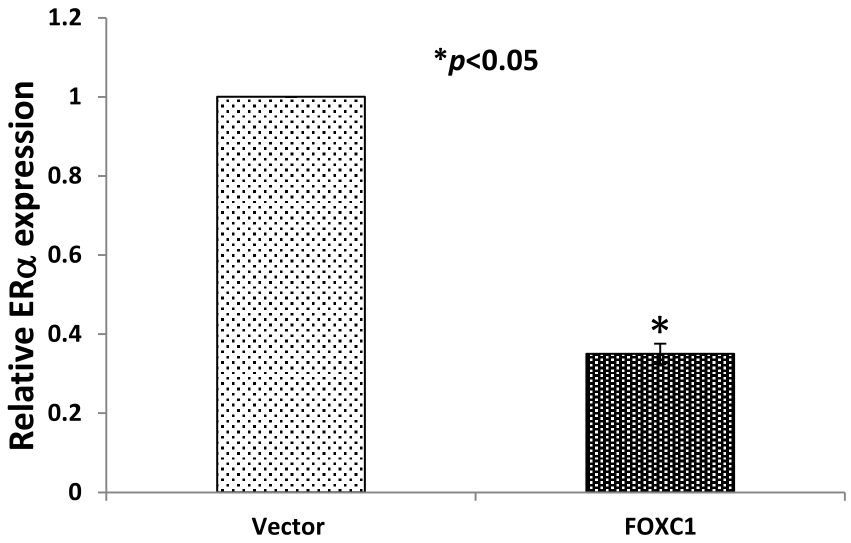

T47D

Supplementary Figure 5

A

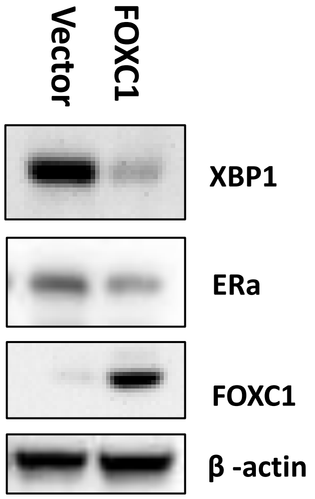

T47D

B

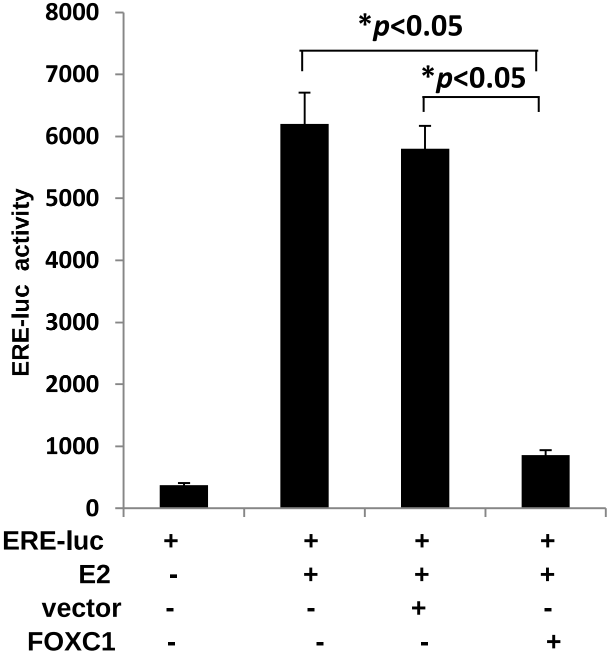

T47D

Supplementary Figure 6

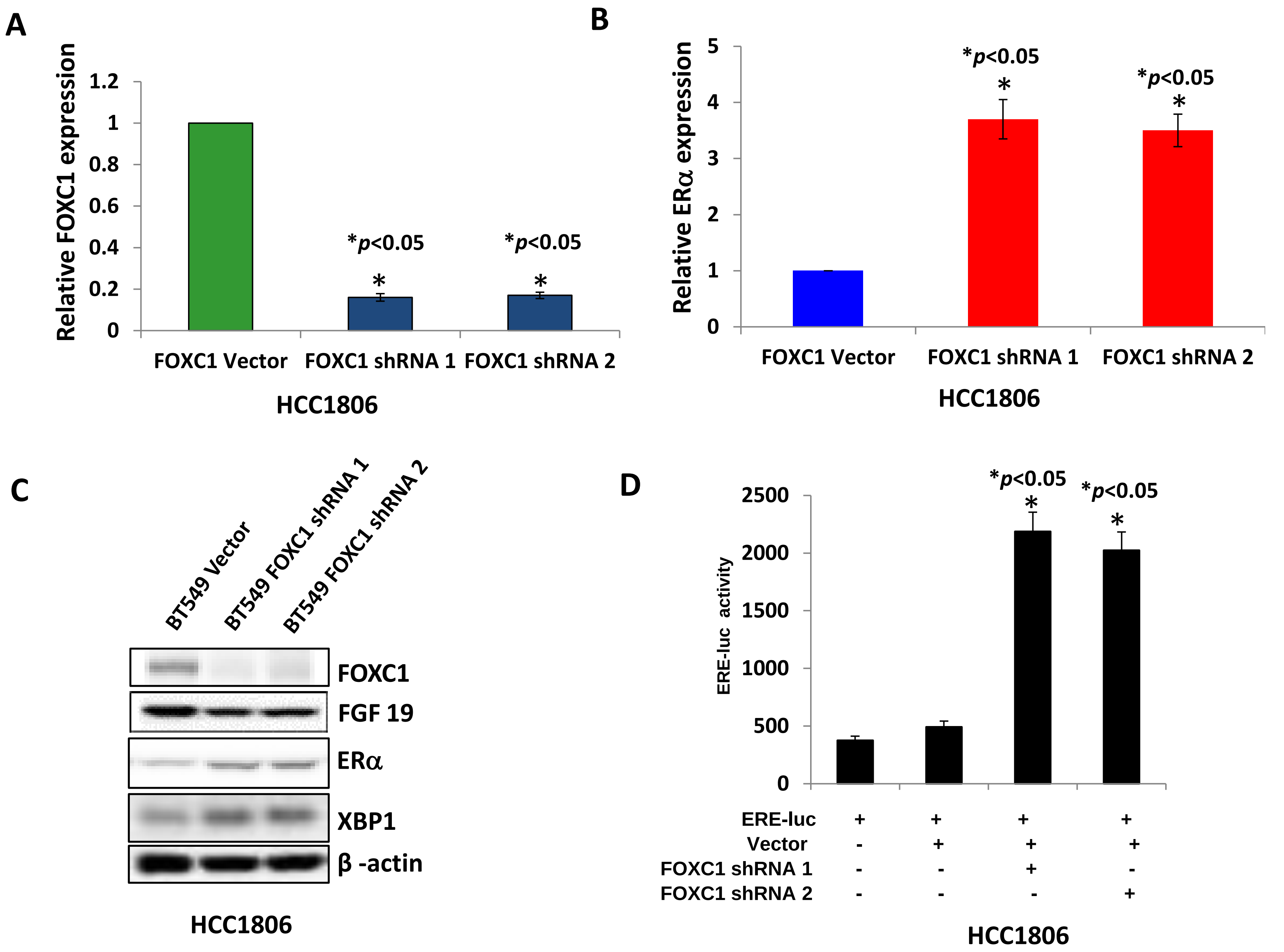

Supplementary Figure 7

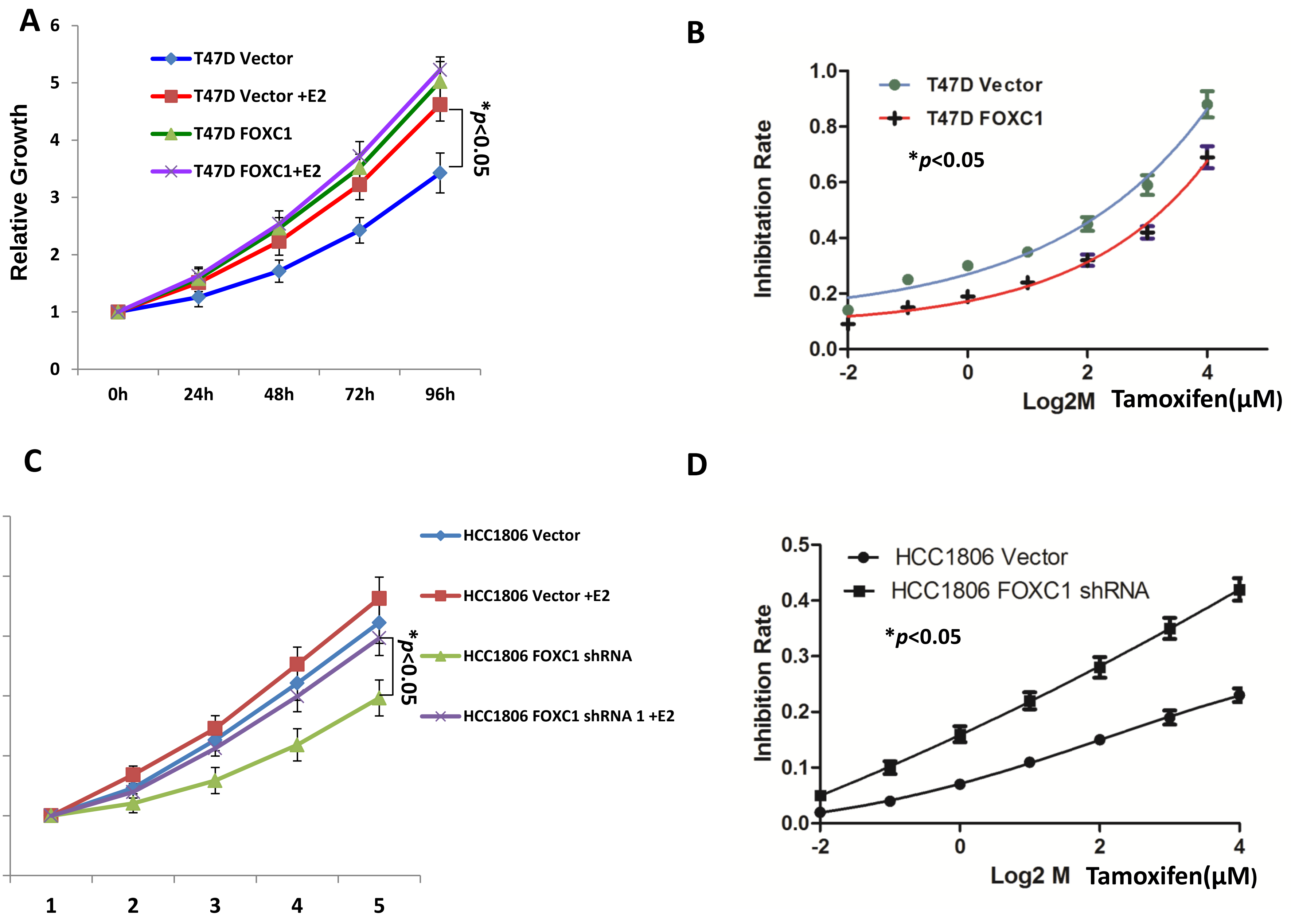

Supplementary Figure 8

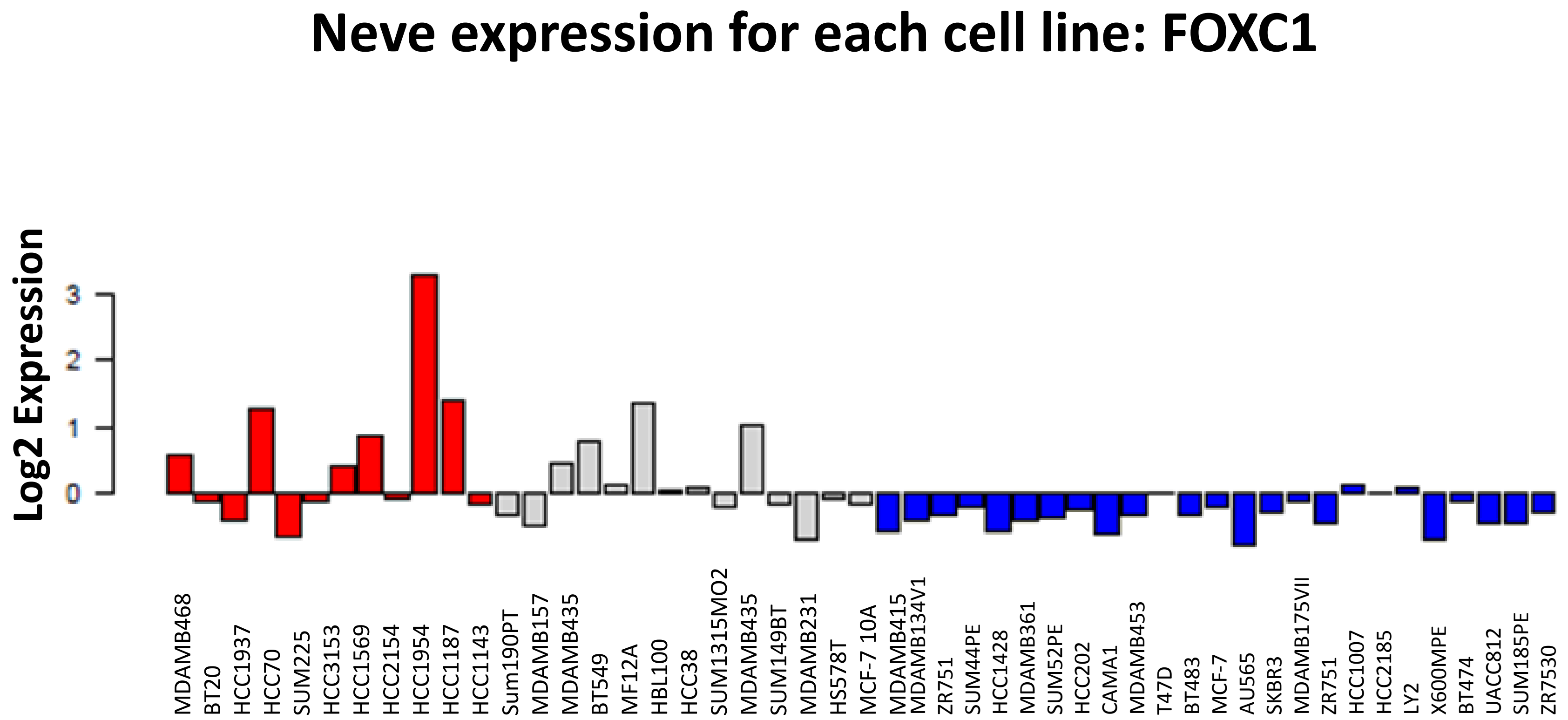

Supplementary Figure 9

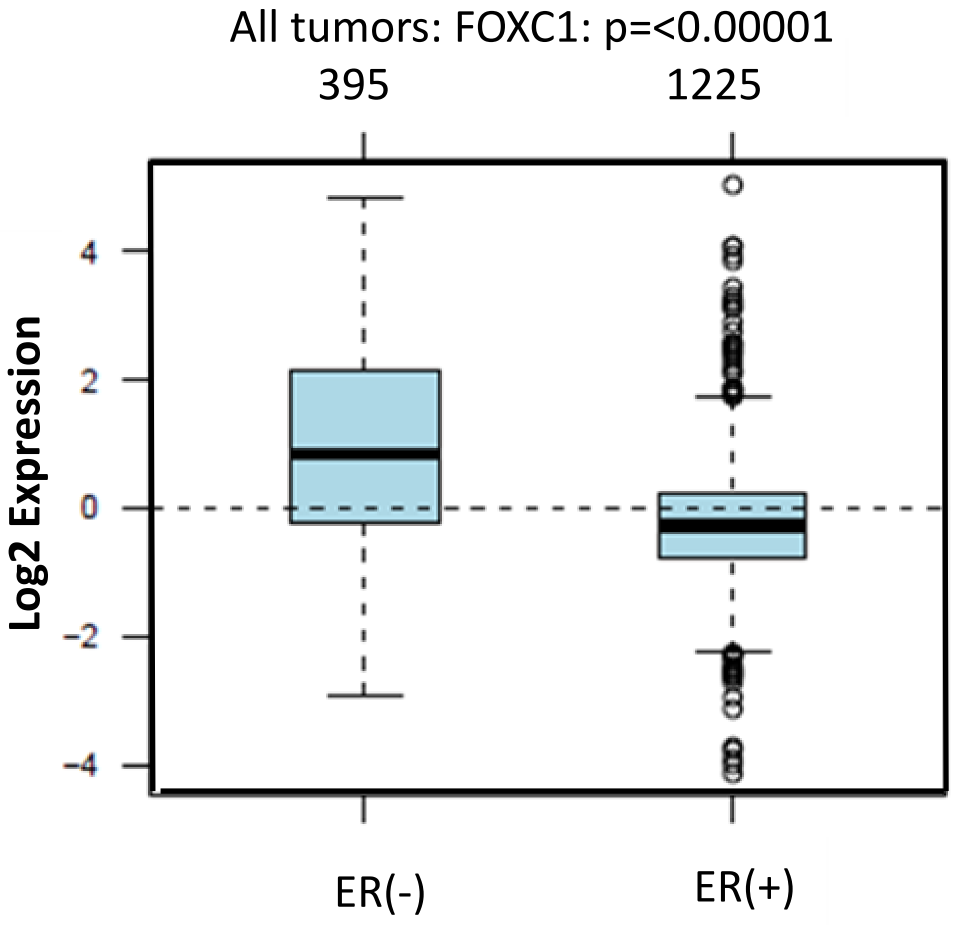

Supplementary Figure 10

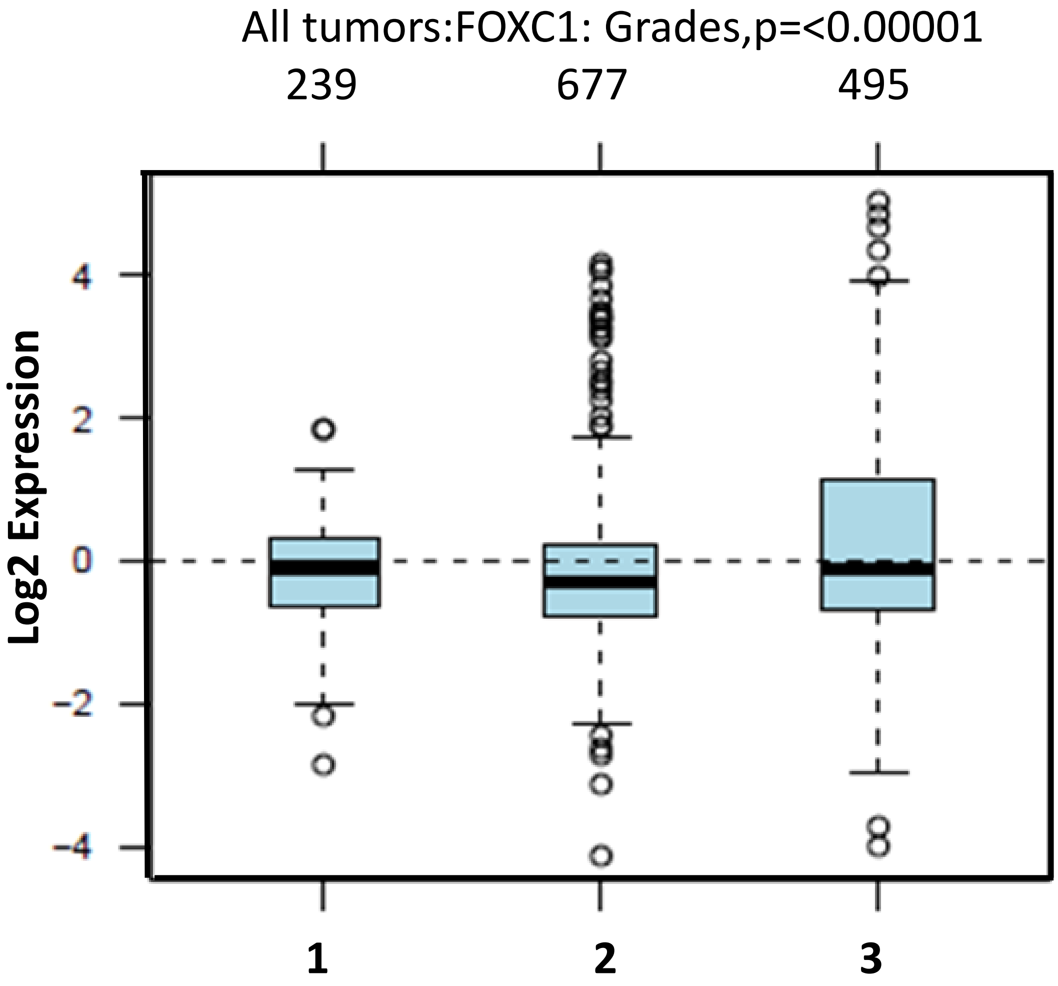

Supplementary Table 1   Primers for RT-qPCR Assays

Biomarker	Forward	Reverse
ER	CGG TTA GAT TCA TCA TGC GGA AC[FAM]G	TGT GTA GAG GGC ATG GTG GAG
FOXC1	CGG TAT CCA GCC AGT CTC TGT AC[FAM]G	GTT CGG CTT TGA GGG TGT GTC
B2-microglobulin	TGTCACAGCCCAAGATAG	CCAGCAAGCAGAATTTGGAA
